# An investigation of the physic-mechanical properties of the expanded perlite mortars with thermal-activated concrete waste and microencapsulated paraffin

**DOI:** 10.1016/j.heliyon.2024.e39720

**Published:** 2024-10-23

**Authors:** Nastasia Saca, Lidia Radu, Răzvan Calotă, Raul-Augustin Mitran, Cosmin Romanițan, Roxana Truşcă, Carmen Răcănel, Ionuț Radu

**Affiliations:** a Technical University of Civil Engineering, Faculty of Roads, Railways and Bridges, Romania; b Technical University of Civil Engineering in Bucharest, Building Services Faculty, Romania; c “Ilie Murgulescu” Institute of Physical Chemistry, Romania; d National Institute for Research and Development in Microtechnologies-IMT-Bucharest, Romania; e National University of Science and Technology Politehnica of Bucharest, Faculty of Engineering in Foreign Languages, Romania

## Abstract

In the transition to zero waste and sustainable development, it becomes essential to use phase change materials and recycled cement in construction projects to improve energy efficiency and encourage sustainable building practices. The primary goal of this study is to determine how the properties of expanded perlite mortars are affected when Portland cement is partially replaced with recycled cement, produced by thermally treating concrete waste at 550 °C. Recycled cement substituted Portland cement in various percentages (10 %, 30 %, and 50 %). Also, microencapsulated phase change material (m-PCM) was incorporated into the mortar in a proportion of 2 wt% and 5 wt%. Cement-based mortars with a 1:3 aggregate volume ratio have been developed for evaluation. The mortars were analyzed in terms of mechanical strength (flexural and compressive), water absorption, thermal properties, differential scanning calorimetry, and microstructure. The experimental results revealed a decrease in mechanical strength values when Portland cement is substituted by recycled cement, regardless of percentage. Recycled cement in proportion of 30 % positively influenced the water absorption. The thermal conductivity of reference mortar was higher than that of 30 % recycled cement. The reference mortar's specific thermal conductivity values were 0.466 W/(m·K) at 25 °C, 0.525 W/(m·K) at 30 °C, and 0.589 W/(m·K) at 35 °C. On the other hand, the mortar containing 30 % recycled cement exhibited much lower heat conductivity throughout these temperatures, suggesting superior insulating qualities. DSC analysis verified that adding m-PCM to the mortars increased their thermal storage capacity, which is essential for raising the energy efficiency of building materials. Nevertheless, the mechanical strength decreased as m-PCM was added. Despite decreases, all mortars satisfied the SR EN 998–1 standard's CS IV classification for interior plaster applications, which is based on compressive strength requirements. Overall, this study shows how using m-PCM and recycled materials can improve the performance of cement-based materials.

## 1 Introduction

This study centers on the dual challenge of managing construction and demolition waste, which constitutes over one-third of total waste in the European Union [1], and the need to develop energy-efficient building materials that contribute to reduced energy consumption in buildings. This issue is critical as the construction industry seeks to balance environmental sustainability with the growing demand for buildings that are not only structurally sound but also energy efficient.

The importance of this study for real-world applications lies in its focus on the valorization of construction waste - specifically, concrete waste - through its use as a partial substitute for Portland cement in mortar production. Traditional Portland cement production is energy-intensive and a significant source of CO₂ emissions, so finding ways to incorporate recycled materials like concrete waste into new construction materials directly addresses these environmental concerns [[2], [3], [4], [5], [6], [7]]. Moreover, the study outlines the limitations of using recycled concrete in mortars, particularly the reduction in mechanical strength observed when the recycled content exceeds 30 % [8,9]. To obtain sustainable construction materials with recycled concrete aggregates were elaborated studies on the behavior of concrete reinforced with steel fibers and polypropylene fibers and variation of its mechanical characteristics using in-situ 4D CT experiments [10,11]. The potential use of thermally treated concrete waste as recycled cement is being extensively studied due to the partial recovery of concrete properties after fire. Florea et al. [12] found no significant strength loss when replacing up to 20 % of cement with untreated or 800°C-treated concrete waste and up to 10 % fines with 500°C-treated waste. Kim et al. [13] observed that replacing 20 % of cement with recycled concrete reduced mortar drying shrinkage by 10 %. Regarding microstructure, Shui et al. [14] studied the rehydration capacity of preheated recycled concrete, finding a looser microstructure in the rehydration products. AFm phase formation and reduced strength during rehydration of recycled cement thermally treated at 700 °C were reported by Bogas et al. [15]. This makes it essential to find innovative solutions that mitigate these drawbacks while enhancing other properties, such as thermal performance.

The proposed solution aligns with the problem stated above by integrating microencapsulated Phase Change Materials (PCMs) into recycled cement mortars, specifically expanded perlite mortars. PCMs can absorb, store, and release heat during phase transitions, which significantly enhances the thermal performance of building materials [[16], [17], [18]]. This improvement is crucial in developing energy-efficient buildings that can maintain stable indoor temperatures with reduced reliance on external heating and cooling systems. The inclusion of expanded perlite, a lightweight and porous aggregate, further supports the storage and distribution of PCMs within the mortar, adding to the material's overall energy efficiency [[19], [20], [21]].

While the addition of PCMs does result in some reduction in mechanical strength, this is offset by the significant gains in thermal performance, making the material particularly suitable for non-load-bearing applications such as interior plaster [22]. The study, therefore, contributes to the development of building materials that are not only sustainable by reducing waste and carbon emissions but also practical for real-world applications where energy efficiency is a priority.

Both inorganic and organic PCMs have issues like corrosiveness and flammability, respectively [23,24]. Paraffin-based PCMs are inexpensive but non-renewable and flammable, whereas bio-based fatty acids are safer and renewable but costlier [25]. Direct incorporation risks PCM leakage in the melted state, which can be mitigated through microencapsulation, macroencapsulation, shape stabilization, and porous inclusion [26,27]. Microencapsulated PCMs are commonly used in building-integrated thermal storage, but research continues to improve their mechanical and thermal properties, with new encapsulation techniques and matrices being explored alongside advanced computational methods to determine their thermophysical parameters [28].

Research into integrating PCMs in construction materials includes experimental studies and modeling their interaction with building structures. The potential for recycling these materials at the end of a building's life is also crucial, as it complements life cycle assessments and ensures compliance with European sustainability standards [29,30].

Elnajjar [31] studied the thermal performance of bricks with organic PCM in hot climates, finding that PCM reduced heat load fluctuations and cooling energy consumption, lowering greenhouse gas emissions. Castellon et al. [32] demonstrated that incorporating PCMs improves thermal comfort and reduces energy use without significantly increasing material weight. Beltran et al. [33] used Building Energy Simulations to evaluate PCMs for wallboards and roofs, identifying paraffins RT25 and RT30 as top performers in Ecuador's climate. Hakim et al. [34] showed that beeswax PCM in Indonesian building materials lowers inner wall temperatures and reduces air conditioning energy consumption. Cunha et al. [35] found that PCM mortars delay temperature fluctuations and reduce HVAC costs, with gypsum mortars providing the best thermal regulation due to high microporosity. Rehman et al. [36] reported a 54.3 % reduction in indoor temperatures using RT28HC and RT21HC paraffin in concrete and cement blocks.

Expanded perlite enhances PCM storage integrity due to its porous structure [[37], [38], [39]]. The porous particles also improve heat transfer efficiency, creating durable, energy-storing composite materials [40]. Building materials with expanded perlite meet heat storage standards and offer financial benefits through increased energy efficiency. Methods for incorporating PCMs include direct mixing, impregnation of porous materials, and micro or macro encapsulation [41,42]. Impregnating lightweight materials like perlite, diatomite, and bentonite is more practical and cost-effective than microencapsulation [[43], [44], [45]]. The main challenge with paraffin and lightweight aggregates is PCM leakage, which shape-stabilized PCMs can diminish.

This study contributes to the ongoing research in developing multifunctional building materials that are in line with the increasing demand for energy efficiency. While higher PCM content does reduce compressive strength, experimental evaluation, and mathematical modeling are used to establish if the trade-off in thermal performance is significant and valuable, especially in climates where temperature regulation is critical.

The authors' research aligns with the current advancements in the field. It makes significant contributions by examining the effects of partially substituting cement with recycled cement and incorporating microencapsulated paraffin on the mechanical and thermal performance of perlite mortars. The study investigated three percentages of cement substitution (10 %, 30 %, and 50 %), while also adding encapsulated paraffin in proportions of 2 wt% and 5 wt %.

## 2 Materials and methods

### 2.1 Materials

**Fig. 1:**
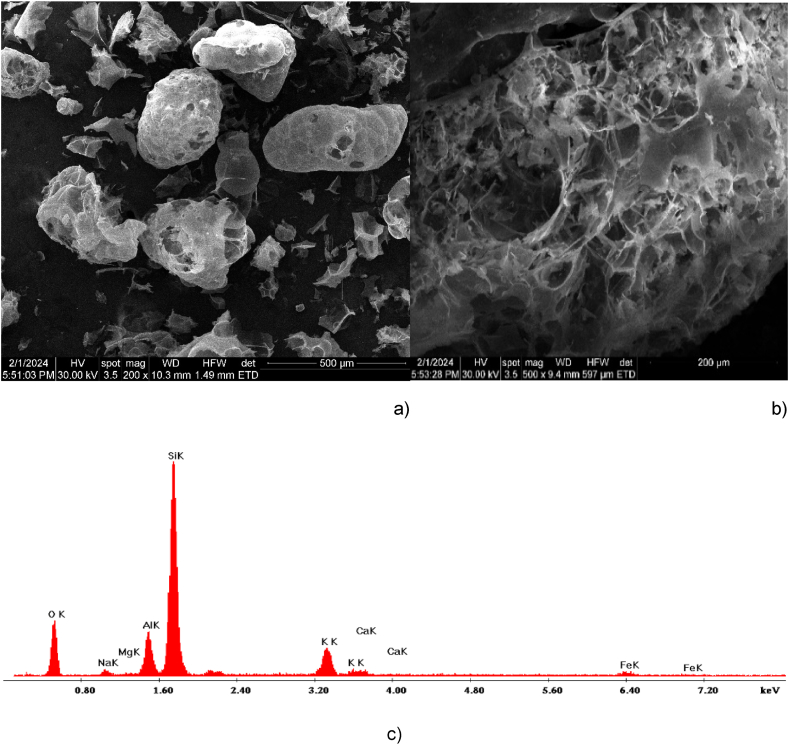
Microstructure (SEM) of a perlite bead -a), b) and EDAX analysis -c).

This work used expanded perlite particles with sizes smaller than 2 mm (Table 1). The SEM images of perlite reveal a foam-like cellular structure (Fig. 1). It is evident from Fig. 1 a) and b) that expanded perlite comprises many randomly shaped particles that mimic flower petals. The main component of the expanded perlite sample depicted in Fig. 1 c) according to the EDAX analysis is silica. Furthermore, traces of several metals, including calcium, oxygen, aluminum, magnesium, potassium, and iron can be observed.

**Table 1:** Properties of perlite.

Colour	Light grey
Bulk density (SR EN 1097–3/2002)	40–65 kg/m^3^
Size distribution (SR EN 933/2002)	0–2 mm, max 10 % 0.5 mm
Thermal conductivity coefficient (SR EN 12667/2002)	max 0.042 W/m K
Compaction resistance (SR EN 13055-1/2003)	Min. 0.07 N/mm^2^
Fire reaction class	A1

The cement type CEM II/A-LL 42.5 R (Heidelberg Materials) was considered a reference. The cement was partially replaced with thermal-treated concrete waste to obtain the mortars.

**Fig. 2:**
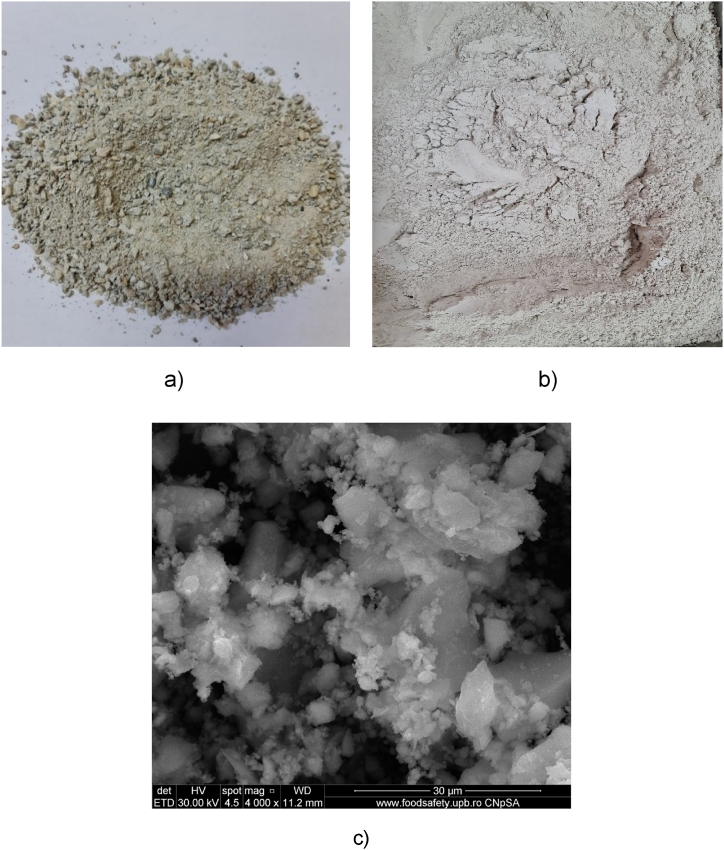
The images of concrete waste in different stages of obtaining: a) original concrete waste under 4 mm; b) concrete waste after thermal treatment; c) SEM image of C-tt.

The concrete with the age of 6 months (prepared in the laboratory, grade C30/37) was crushed with a jaw crusher and sieved until the material was smaller than 4 mm (Fig. 2). Thermal treatment of materials was done in an electric oven Nabertherm N11/H at 550 °C, for 3 h, with a heating rate of approximately 20 °C min^−1^, maintaining the highest temperature for 3 h. The product was allowed to cool in the oven until room temperature and named C-tt. After cooling the materials were kept in the oven at 80 °C until use. During the thermal treatment, hydrates lose water (between 20 and 130 °C), gypsum and ettringite dehydrate (110 and 200 °C), C-S-H and calcium carboaluminate hydrates dehydrate (140 and 450 °C), and Portland dihydroxylation occurs (450 and 650 °C) [46,47]. The irregular shapes of C-tt may be observed in Fig. 2.

The characteristics of thermal-treated concrete and reference cement are presented in Table 2.

**Table 2:** Characteristics of calcined materials and cement CEM II/A-LL 42.5R

	Thermal-treated concrete waste (C-tt)	CEM II/A-LL 42.5R (R)
*Bulk density (kg/m*^*3*^*)*	*782*	*1113*
*Water for standard consistency (%)*	*0.36*	*0.31*
SR EN 196-3		
*Setting time (min.), SR EN 196-3 initial*	250	135
final	480	*195*
*Compressive strength (MPa) on paste, 28 days*	*10.6*	58.3

#### 2.1.1 Phase change material

**Fig. 3:**
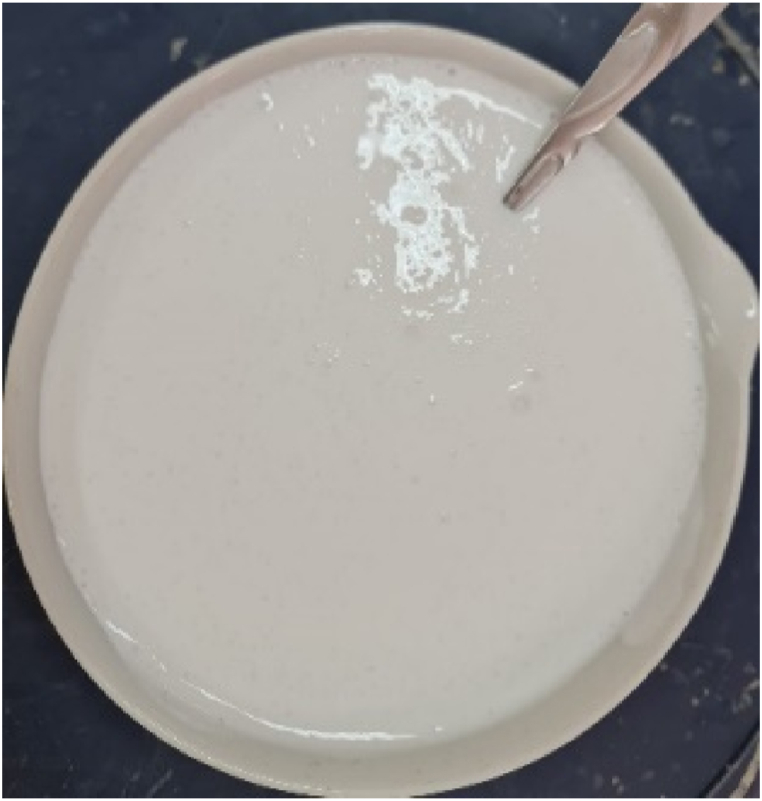
Image of m-PCM mixed with water.

Microencapsulated paraffin wax slurry (m-PCM) was used as the phase change material. The characteristics of the material, according to the producer are: type of membrane: melamine-formaldehyde; PCM content in the dispersion: 25–30 %; PCM content in the dry capsule: 75–80 %; phase change temperature: 22–26 °C; heat storage capacity>160 J/g, pH 7–9; density 900–970 kg/m^3^; average particle size: 1–20 μm. When thermal comfort is the goal, the PCM melting temperature range for their integration in buildings’ walls is in general between 20 and 28 °C, therefore, consideration of this crucial factor must be given when selecting these materials [48]. Air bubbles were observed at the surface when m-PCM was slightly mixed with water (Fig. 3).

### 2.2 Methodology

The experimental program examined how calcined concrete waste and m-PCM affect expanded perlite mortar's characteristics. Seven mixes were used in the experimental study: one reference mortar (R), three mortars with Portland cement partially substituted by calcined concrete waste, and three mortars with varying PCM levels (2 % and 5 %) or types of cement (cement was partially substituted by 30 % C-tt). The mortar compositions are displayed in Table 3. For all mortar mixes, laboratory-prepared mortar samples were utilized with a constant ratio cement: expanded perlite of 1:3. Water/cement ratio was modified for each mix to obtain a mortar with consistency between 140 mm and 200 mm. In the case of mortars with m-PCM, the phase change material was mixed with approximately 60 % water and added into the mix composed of cement paste and expanded perlite. After mixing the mortars were tested for consistency. After that, the mortars were placed in 40 mm × 40 mm x 160 mm and 60 mm × 300 mm x 300 mm prism-shaped molds and kept in the molds for 24 h, covered with polyethylene film, in a laboratory atmosphere. The samples were cured at 20 °C and a relative humidity of 95 ± 5 % until testing time. In addition, the mortars were tested for flexural and compressive strength, water absorption by capillarity, and thermal performance. In addition, microstructure (SEM) and elemental analysis (energy dispersive X-ray spectroscopy EDAX) were also performed.

**Table 3:** Composition of mortars (kg/m^3^).

Mortar code	Perlite (kg)	CEM II/A-LL 42.5R (kg)	C-tt (kg)	PCM (kg)	Water/cement	Consistency (mm)
R	176.5	852.4	–	–	0.53	142
C10	153.8	678.9	75.5	–	0.63	155
C30	154.2	529.2	226.8	–	0.63	150
C50	145.9	356.5	356.5	–	0.66	157.5
R2	153.7	751.8	–	15.1	0.55	141
R5	157.2	769.6	–	38.5	0.56	140
C30-2	149.5	512.5	219.6	11.7	0.65	152

Notably, the total volume of water recorded in Table 3 did not include the amount of water coming from m-PCM.

### 2.3 Methods

#### 2.3.1 Mortars in the fresh state

*The consistency* of mortars was tested by the flow table method [49]. Two layers of mortar were added to the truncated mold, and each layer was tamped ten times to guarantee uniform filling. The extra mortar was removed by cutting it off, and the mold was removed. The table was dropped fifteen times right away. A caliper was used to measure the average diameter.

#### 2.3.2 Mortars in the hardened state

##### 2.3.2.1 Water absorption

After 28 days, three mortar samples were dried at 105 °C in an oven until a constant weight, m_i_, was achieved. According to SR EN ISO 15148:2004 [50], the specimens were sealed to limit the passage of water to one face using a hydrophobic coating after being stored in a desiccator until they reached room temperature. The samples were submerged in water to a depth of 5 ± 2 mm, with a constant water level. The weight of the mortars was measured at 20, min, 1 h, 2 h, 4 h, 8 h, and 24 h following immersion, and their mass, m_t_, was determined. The increase in mass reported to the surface of samples in contact with water, A, was calculated with Equation (1):

(1) $$\begin{array}{cc} \Delta m_{t}=\frac{m_{t}-m_{i}}{A}\;\left(\text{kg}/m^{2}\right) & W=\frac{m_{t}-m_{i}}{A}\;\left(kg/m^{2}\right) \end{array}$$

##### 2.3.2.2 Mechanical strength

The compressive strength of the mortars was assessed at 7 and 28 days. Three specimens measuring 40 x 40 × 160 mm^3^ were examined at each age to determine the average compressive and flexural strength [51]. The samples were positioned perpendicular to the casting direction before loading. All samples were tested using hydraulic testing equipment with 300 kN and 3000 kN load capabilities.

##### 2.3.2.3 Thermal conductivity

The thermal conductivity testing of the samples was conducted using a hot plate-cold plate setup, according to the procedure outlined in EN 12667:2001 [52]. Samples, pre-formed into 30 × 30 cm sections, were assessed for their thermal response. The system monitored the temperatures and heat flux between the plates until a steady state was achieved after approximately 4 h. This ensured full phase transition under the stable thermal gradient. Thermal conductivity was then calculated based on the steady-state data, allowing for accurate evaluation in line with standards for high and medium thermal resistance building materials.

##### 2.3.2.4 X-ray diffraction

X-ray diffraction investigations were performed to identify the crystalline phases in anhydrous and hydrated Portland cement/recycled cement. In this scope was used a 9 kW Rigaku SmartLab diffractometer equipped with a monochromatic CuKα1 source that provides a wavelength, λ = 0.15406 nm. with Ni-filtered Cu Kα radiation (λ = 1.5406 Å), with a 2θ range of 5–60°, a scanning speed of 5°/min, and a step size of 0.01°.

##### 2.3.2.5 Differential scanning calorimetry (DSC)

A DSC analysis was performed on m-MSF and mortars to evaluate their potential for storing thermal energy. One of the most significant thermal properties of PCMs is enthalpy as a function of temperature. For this reason, it is essential to conduct thermal analysis of the PCM melting and crystallization processes to determine the phase change temperature range and the enthalpy as a function of temperature. Differential scanning calorimetry (DSC) analyses were performed using a Mettler Toledo DSC 3 calorimeter, at a scanning rate of 10 °C min^−1^, under 80 mL min^−1^ nitrogen flow. In situ optical microscopy (OM) was carried out during the DSC measurements using a SC50 Olympus microscope, at a frame rate of 1 image per °C.

##### 2.3.2.6 Microstructure of mortars

Scanning electron microscopy (SEM) combined with energy-dispersive X-ray spectroscopy (EDAX) is a technique used to obtain images of the surface of mortars and establish the elemental composition of samples. The microstructure of materials and 28-day mortars was examined by SEM analysis using an Inspect F 50 Scanning Electron Microscope fitted with an Energy Dispersive X-ray spectrometer. The samples were given a small layer of gold, a conductive coating, before imaging.

## 3 Results and discussion

### 3.1 Characterization of concrete waste

**Fig. 4:**
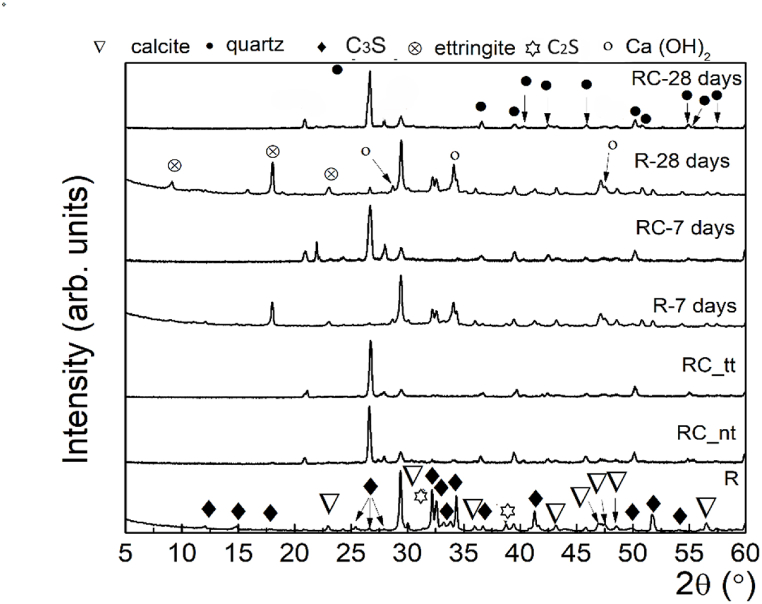
XRD patterns of anhydrous Portland cement (R), Portland cement hydrated for 7 days and 28 days (R-7 days and R-28 days), concrete waste before thermal treatment (RC_nt) and after thermal treatment (RC_tt) and hydrated for hydrated for 7 days and 28 days (RC-7 days and RC-28 days).

The composition of Portland cement and concrete waste underwent X-ray diffraction investigation to describe it. The comparative XRD patterns displayed in Fig. 4 indicate that quartz and calcite are the primary constituents of concrete waste. The carbonization of the concrete over time may be the cause of the calcite's presence. C_3_S and C_2_S were all present in the untreated concrete. One cement/concrete waste calcination product that was not identified was calcium oxide. The comparison between untreated and calcined concrete waste led to two observations. By analyzing the diffraction peaks of the Ca (OH)_2_, was observed that the diffraction peak from 2θ = 34.16° disappeared for calcined concrete waste (RC_tt), which indicates that the compound was decomposed during the thermal treatment of concrete waste. Also, by fitting the main quartz peak (2θ = 26.75°) with a pseudo-Voigt fit before and after thermal treatment, it was found that the Full Width at Half Maximum (FWHM) is preserved, an FWHM ∼ 0.23° being reported in both cases. Thus, one can infer that the temperature did not affect the quartz crystallinity. However, a small lattice strain of the quartz occurred after thermal treatment, as revealed from the peak position.

As hydration products, ettringite and calcium hydroxide were determined for both materials, at 28 days. Calcium silicate hydrate is amorphous and undetectable by XRD analysis.

The water for standard consistency was higher for C-tt than for the reference cement (Table 2). At the setting time, a stiffening of the C-tt paste was observed after 250 min, compared to 135 min for R. These results are correlated to lower activity of C-tt compared to CEM II/A-LL 42.5 R cement as XRD analysis reveals and its structure. A similar result was obtained by S. Real et al. [53]. The longer setting time of C-tt than Portland cement may be caused by the fact that CaO was pre-hydrated and the lower content of cement in concrete waste than in cement paste. The CaO was not found, even though Ca(OH)_2_ should have dehydroxylated at the treatment temperature.

**Fig. 5:**
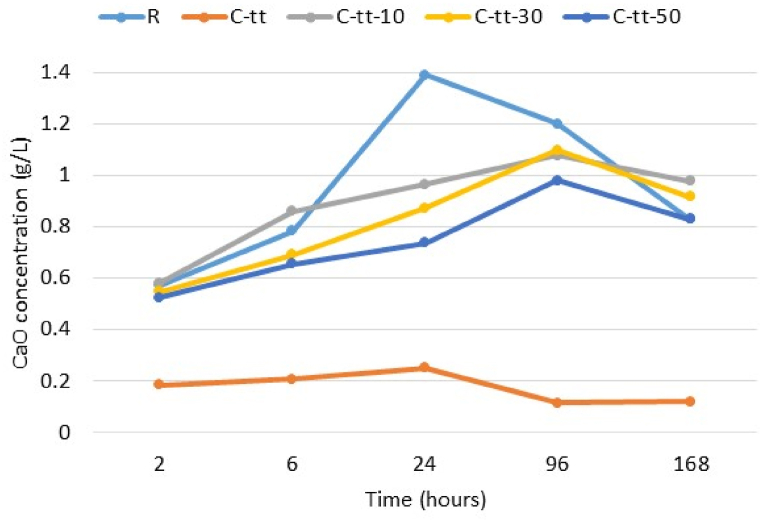
Variation of CaO concentration in suspensions over time.

Suspensions (liquid/solid ratio of 50/1) were prepared to see the effect of C-tt on the pH and CaO concentration at 2 h, 6 h, 24 h, 96 h, and 168 h. The reference cement was substituted by 0 %, 10 %, 30 %, 50 % and 100 % wt. C-tt. The pH measured with Jenway 3540 is presented in Table 4. The pH of C-tt suspension was lower than that of reference and blended cement, in correlation with the low content in calcium silicate phases revealed by the XRD analysis. The variation is discontinuous and correlated to the evolution of the hydration process (Fig. 5). The Ca^2+^ ions assessed based on complexometric titration (murexide indicator) had also a discontinuous variation. The maximum concentration of CaO was reached after 24 h, for R and C-tt, and after 96 h for the others.

**Table 4:** pH of suspension.

Time (hours)	pH				
	R	100 % C-tt	90%R+10%C-tt	70%R+30%C-tt	50%R+50%C-tt
2	12.42	11.9	12.62	12.56	12.49
6	12.63	11.96	12.75	12.69	12.5
24	12.71	12.19	11.56	12.6	12.63
96	12.83	12.07	11.96	11.97	12.01
168	12.83	12.08	12.54	12.63	12.66

The reference cement is the type of CEM II/A-LL 42.5, which shows limestone addition. The localized growth of Ca(OH)_2_ into massive crystals, can be prevented by CaCO_3_, which can adsorb the Ca^2+^ ions produced during cement hydration. This adsorption promotes the preferred nucleation of Ca(OH)_2_ particles surrounded by CaCO_3_.The fine CaCO_3_ particles lower the activation energy and enhance the matrix's hydration process by serving as C-S-H nucleation sites [54]. Carbonate aluminate hydrates are also generated by the reaction between calcium carbonate and C_3_A and C_4_AF, which leads to an increase in the volume of hydration products and enhances the structure of the matrix and mechanical strength values [55].

### 3.2 Workability of mortars

The flow table test was used to evaluate mortars' workability, following the SR EN 1015-3 standard [49]. The measurements of expanded perlite mortar with cement partially substituted by C-tt and mortar with m-PCM are presented in Table 3. The consistency of mortars with C-tt varies from 150 mm to 157.5 mm. These values are higher than that of the reference cement. An increase in the water content of mortars was obtained due to C-tt particles. The values of flow diameter were 141 mm for R2 and 140 mm for R5 compared to 142 mm for R. An increase in water/cement ratio was also obtained for these mortars with m-PCM. The smaller microcapsules in mortar can have ball-bearing effects during the mixing stage [56], as smaller microcapsules help make the mortar more workable. The solid-state nature of PCMs makes them a non-absorptive addition, which raises the PCMs containing mortar flow table values [57].

### 3.3 DSC characterization of the m-PCM and mortars

**Fig. 6:**
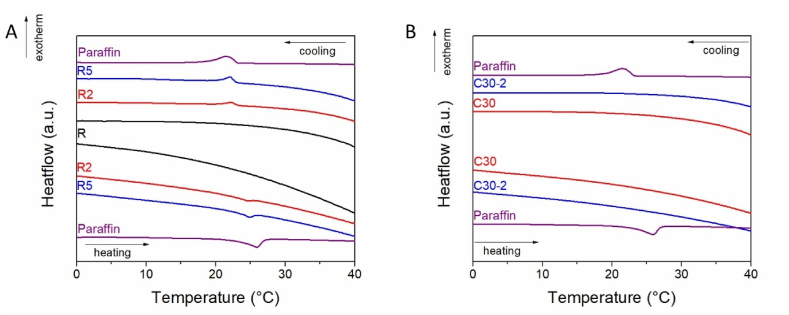
DSC analyses of m-PCM and mortars R, R2, and R5 -a) and mortars C30 and C30-2 – b).

**Fig. 7:**
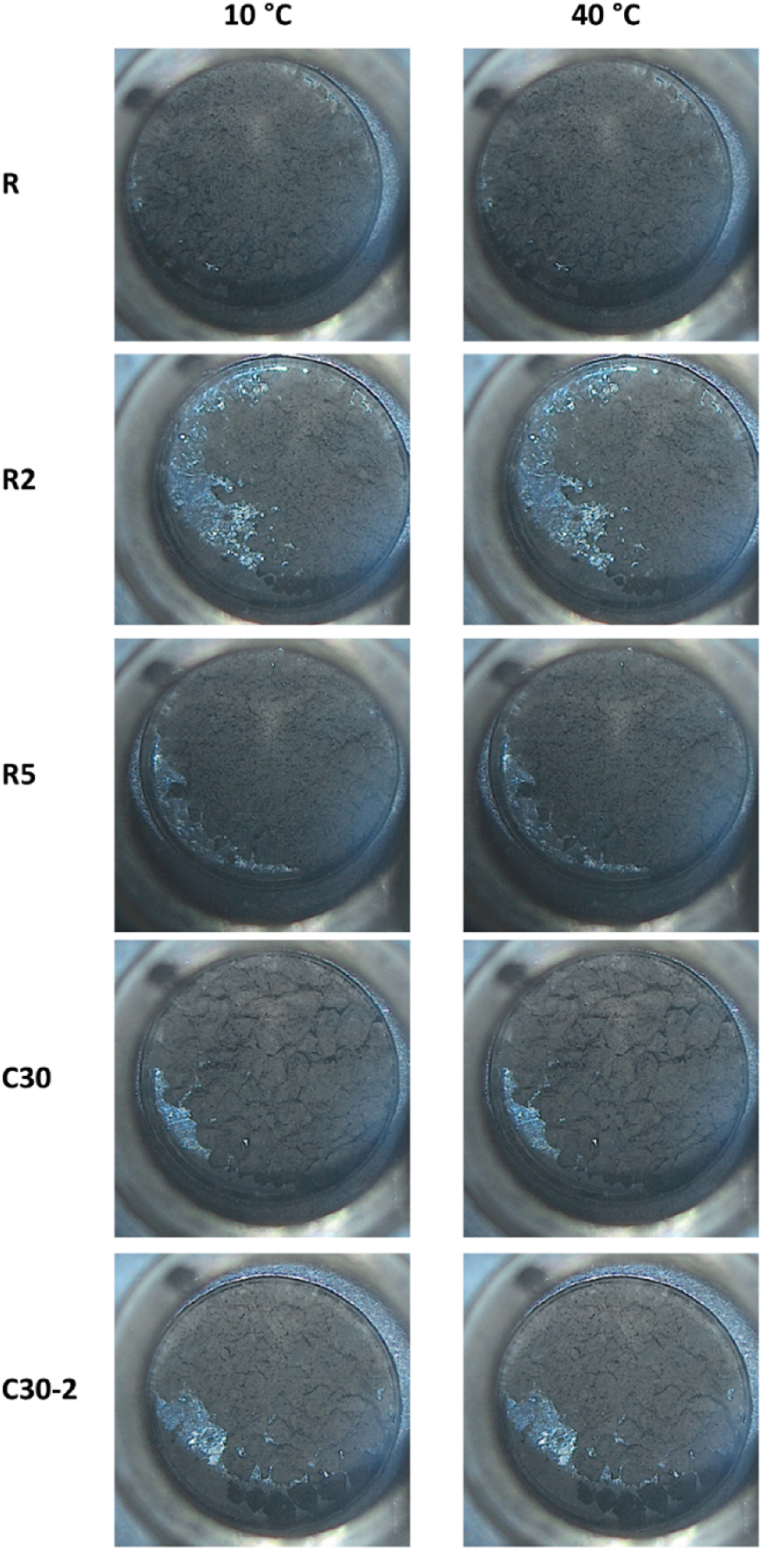
Optical microscopy images of the composites at 10 °C and 40 °C.

DSC analyses were carried out on the mortar samples and paraffin to characterize their thermal energy storage potential (Fig. 6). The DSC analysis of the paraffin shows an endothermic melting event during heating and a reversible, exothermic freezing event during cooling (Fig. 6a). The paraffin has a melting point (m.p.) of 22.7 °C and a freezing point (f.p.) of 23.3 °C, while the heat of fusion of the paraffin is 34.2 Jg^-1^ (Table 1). The PCM content in the commercial microcapsules is around 21 % wt. The reference mortar shows the same melting and freezing transitions as the pristine paraffin sample (Fig. 7a). The transition temperatures for the composite samples are similar to that of the paraffin, indicating that the organic substance exists as a separate solid phase (Fig. 6a) and b)). The heat of fusion for the R2 and R5 samples are 0.05 and 0.10 Jg^-1^, respectively (Table 5). These values are lower than the theoretical values computed based on the initial amount of added paraffin, at 0.14 and 0.30 % wt. This indicates that most of the paraffin molecules are adsorbed onto the mortar.

**Table 5:** Melting (m.p.) and freezing points (f.p.), the heat of fusion (ΔH) values, and free paraffin amount computed from DSC (Paraffin) for the samples.

Sample	m.p. (°C)	f.p. (°C)	ΔH (Jg^−1^)	Paraffin (% wt.)
Paraffin	22.7	23.3	34.2	
R2	24.3	23.2	0.05	0.14
R5	23.4	23.1	0.10	0.30

Optical microscopy coupled with DSC was acquired to assess the shape stability of the materials before and after the melting of the paraffin (Fig. 7). Shape stability refers to the property of a material containing PCMs to retain its macroscopic solid shape even at temperatures where the heat storage phase is molten. Shape stability is typically attained through encapsulation or attractive forces between a high porosity matrix and the heat storage agent. No significant changes could be noticed in the optical micrographs, indicating that the composites retain their shape even at temperatures above the paraffin m.p.

### 3.4 Water absorption

**Fig. 8:**
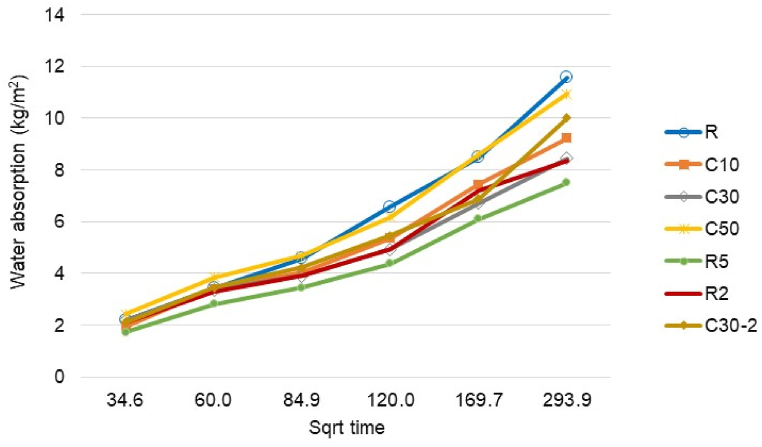
Water absorption of mortars.

The initial water rate of sorptivity via capillarity is displayed in Fig. 8, as a function of the square root of time (Sec1/2). The results show that capillary absorption changes linearly with time (Sec1/2) at the initial sorptivity (R^2^ was between 0.9436 and 0.9683). In general, replacing cement with up to 50 % C-tt positively affected the water absorption of mortars at selected testing times. The experimental results showed a discontinuous decrease in water absorption with C-tt content, the optimal content being 30 %.

Expanded perlite has a unique porous structure. The pore size distribution affects how well mortar absorbs water. Three pore size ranges are present in expanded perlite thermal insulation mortar: 0.01–0.1 μm, 0.1–10 μm, and 10–1000 μm [58]. Free water is adsorbed into the 0.01–10 μm pores [59], while the pores with diameters lower than 10 μm influence capillary water absorption, and those smaller than 0.1 μm control vapor adsorption.

Silva et al. [60] claim that the pores in expanded perlite function as air voids and restrict the capillary action, which means that this aggregate has a far bigger impact on water absorption capacity than absorptivity.

The water absorption decreased when m-PCM was added. The water absorption of R5 was significantly lower than the value of R and R2, regardless of measurement time. For example, the water absorption was 7.5 kg/m^2^ for R5, after 24 h (293.9 sec^1/2^). This value is 10.3 % lower than that of R2 and 35.2 % lower than R. This behavior is explained by the fact that m-PCM is a nonsorptive solid and its addition rerouted capillary flow around the shell. This increased the water flow path's tortuosity and lowered the moisture penetration rate [61]. According to Wei et al. [62], the phase change materials occupy voids and diminish the porous content of the cement mortar. The presence of both m-PCM and C-tt in the mortar increased the water in comparison with R2 and C30. Initially, the water absorption of C30-2 was close to the R2 and C30 values. This difference increased after 2 h (84.9 sec^1/2^). After 24 h, the water absorption of C30-2 was 10 kg/m^2^, compared to 8.36 kg/m^2^ for R2 and 8.44 kg/m^2^ for C30.

### 3.5 Thermal conductivity

**Fig. 9:**
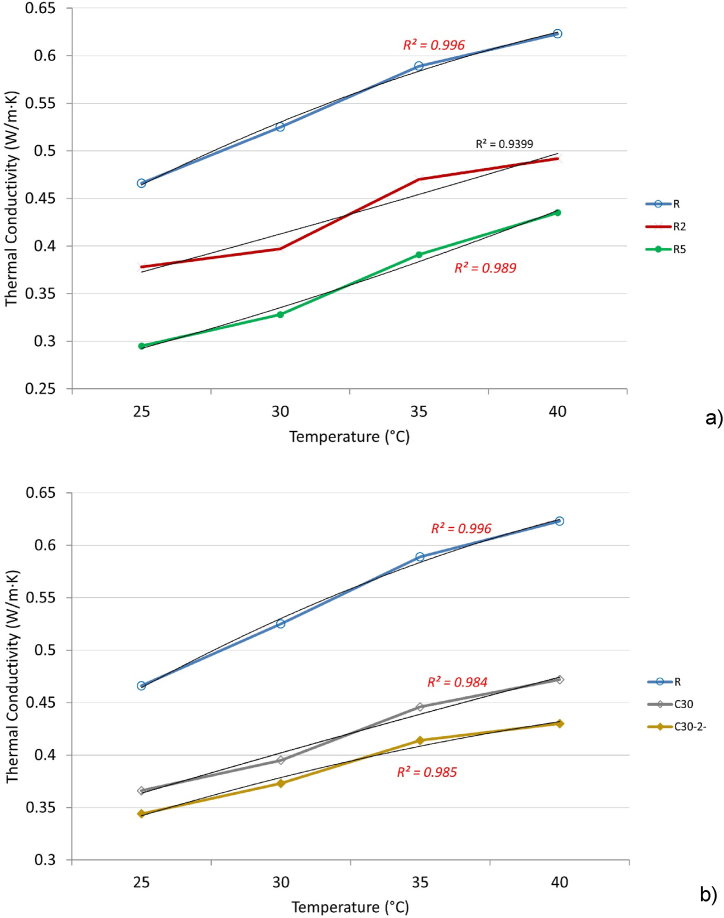
Thermal conductivity as a function of the temperature for the reference mortar and mortars with m-PCM -a); mortars with 30 % recycled cement with/without m-PCM – b).

The density, composition, and air gaps in the mortars all have a direct impact on the thermal characteristics. The many air cells in expanded perlite aggregate provide excellent insulating qualities. Notably, expanded perlite plaster offers up to 4–6 times more resistance to heat transmission than standard sand plaster [63]. The experimental results for thermal conductivity expressed by λ-value, in W/(m·K) are presented in Fig. 9.

From the analysis of both graphs, may be observed that regardless of the temperature at which the tests were conducted, the thermal conductivity of the reference material (R) shows significantly higher values than the other tested materials. The thermal conductivity values for the reference material R are 0.466 W/(m·K) at 25 °C, 0.525 W/(m·K) at 30 °C, and 0.589 W/(m·K) at 35 °C. For materials where recycled cement (C30 and C30-2) and phase change material (R2 and R5) were added, there is a noticeable trend of increasing thermal conductivity with rising temperature (Fig. 9a). It can be inferred that this increasing trend in thermal conductivity continues beyond the measured temperature range (25–35 °C), both at temperatures below 25 °C and above 35 °C. The measurement limits in terms of temperature are strictly related to the experimental setup, which is directly influenced by the temperature of the water supply. At the beginning of testing, the temperature of the equipment's cold plate coincides with the water supply temperature, which typically ranges between 13 and 15 °C. During the testing process, which involves monitoring the evolution of parameters until reaching a steady-state regime, the temperature of the cold plate increases due to the heat transferred through the tested material from the upper plate. The average testing time for the materials in this study was approximately 4 h.

The addition of phase change materials (PCMs) to the composition decreases the material's thermal conductivity as the content of added PCM increases (Fig. 9b). This phenomenon is attributed to the generally lower thermal conductivity of phase change materials, whether encapsulated or directly integrated into the material structure, which is around 0.2 W/(m·K) according to literature references [64].

These observations highlight the significant impact of temperature on the thermal conductivity of the studied materials and provide essential information for evaluating their thermal performance under various operating conditions.

For each dataset corresponding to a specific material, a second-degree polynomial equation was derived to model the material's thermal conductivity as a function of temperature. The primary criterion for determining the equation's coefficients was achieving an R-squared value greater than 0.9 for the variation, which was highlighted in Fig. 9. Four types of relationships were analyzed: linear, logarithmic, exponential, and quadratic (second-degree polynomial). Among these, the quadratic model provided the most accurate results for estimating thermal conductivity, with a relative error of less than 3 % (Equation (2) … (6)). The results are plotted below.

(2) $$\text{For}\;R:\lambda =-\left(2.5∙10^{-4}\right)∙t^{2}+0.02695∙t-0.05325\;\left[W/\left(m∙K\right)\right]$$

(3) $$\text{For}\;C30:\lambda =-\left(3∙10^{-5}\right)∙t^{2}+\left(9.33∙10^{-3}\right)∙t+0.14915\;\left[W/\left(m∙K\right)\right]$$

(4) $$\text{For}\;C30-2:\lambda =-\left(1.3∙10^{-4}\right)∙t^{2}+0.01443∙t+0.06265\;\left[W/\left(m∙K\right)\right]$$

(5) $$\text{For}\;R2:\lambda =\left(3∙10^{-5}\right)∙t^{2}+\left(6.35∙10^{-3}\right)∙t+0.19525\;\left[W/\left(m∙K\right)\right]$$

(6) $$\text{For}\;R5:\lambda =\left(1.1∙10^{-4}\right)∙t^{2}+\left(2.51∙10^{-3}\right)∙t+0.16105\;\left[W/\left(m∙K\right)\right]$$

### 3.6 Mechanical strength

To assess the influence of C-tt on the mechanical strength of mortars pastes with reference cement and 100 % C-tt were prepared and tested for 28 days. The results revealed a significant decrease of 100 % C-tt paste. The compressive strength was 58.3 MPa for CEM II/A-LL 42.5 R paste, respectively 10.6 MPa for C-tt paste. The flexural strength was 3.7 MPa for CEM II/A-LL 42.5 R paste, respectively 2.4 MPa for C-tt paste. This is because a high C-tt replacement ratio leads the specimen to provide a much smaller amount of cementitious material, which lowers the hydrates responsible for mechanical strength and lowers mechanical strength.

**Fig. 10:**
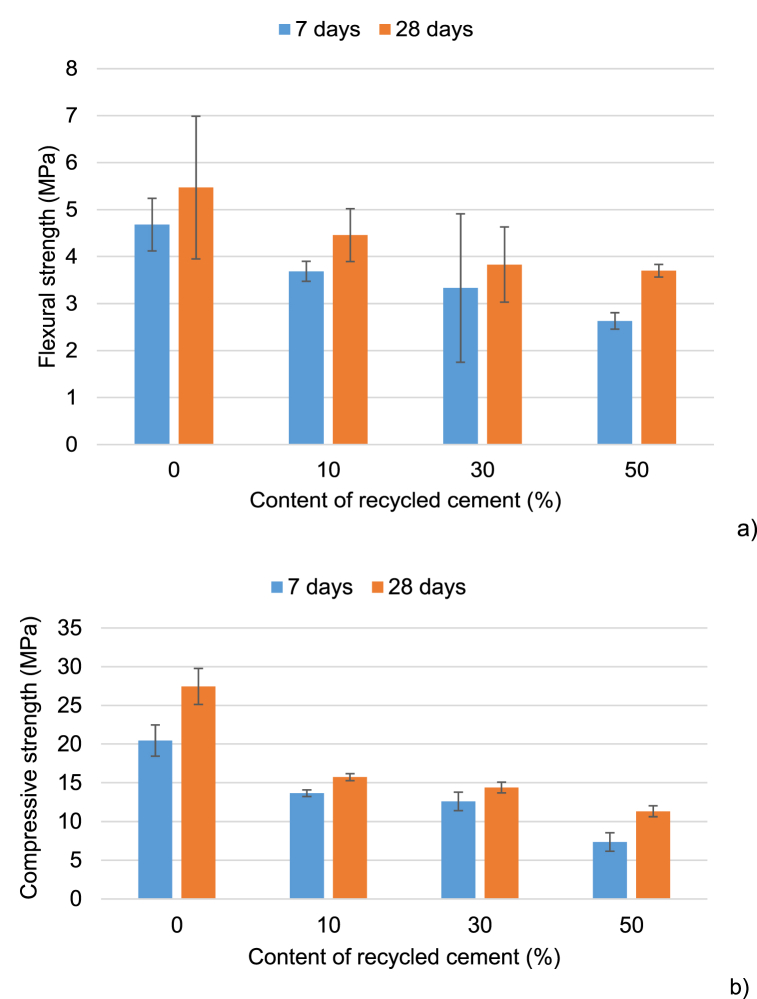
Influence of C-tt content on the flexural (a) and compressive strength (b).

Fig. 10 shows the gradual increase of mechanical strength values with time. The use of C-tt affected the mechanical strength values of the mortars at 7 days and 28 days. The mechanical strength of mortars depends on the cement since expanded perlite has low mechanical strength. The flexural strength of the mortar containing 10 % C-tt (3.56 MPa) reduced after 7 days compared to R (5.47 MPa). The mortars containing 30 % and 50 % C-tt had flexural strength values of 3.7 and 2.63 MPa, respectively. These values represent a decrease of 28.8 % and 43.8 % concerning the reference. The flexural strength of the mortar containing 10 %, 30 %, and 50 % C-tt at 28 days drops by 18.5 %, 30 %, and 32.4 %, respectively. The mortar made entirely of cement showed better flexural strength than mortar containing C-tt at 7 days and 28 days (Fig. 10). This is explained by the fact that the incorporation of C-tt weakens the cementitious characteristics, which lowers the flexural strength of the cement-based mortars. A comparable pattern may be seen in the compressive strength of mortars.

The compressive strength of the reference mortar was 20.5 MPa and 27.5 MPa after 7 days and 28 days, respectively. The C-tt (10 %, 30 %, and 50 %) compressive strength values exhibited a 33.2%–64.1 % decrease in comparison. The compressive strength of the cement containing C-tt (10 %, 30 %, and 50 %) drops by 42.7 % and reaches 58.8 % after 28 days (Fig. 10 (b)). Bogas et al. [65] explained the higher mechanical strength of Portland cement paste compared to recycled cement paste to the formation of a higher volume of interparticle compounds at 28 days, which led to a more compact microstructure.

As Fig. 10 shows the gains in mechanical strength were slow with time (between 0.5 MPa and 1.41 MPa for flexural strength and in the range of 0.2 MPa … 4 MPa for compressive strength) associated with the composition of recycled cement and low rates of hydrates formation as revealed XRD patterns (Fig. 4).

**Fig. 11:**
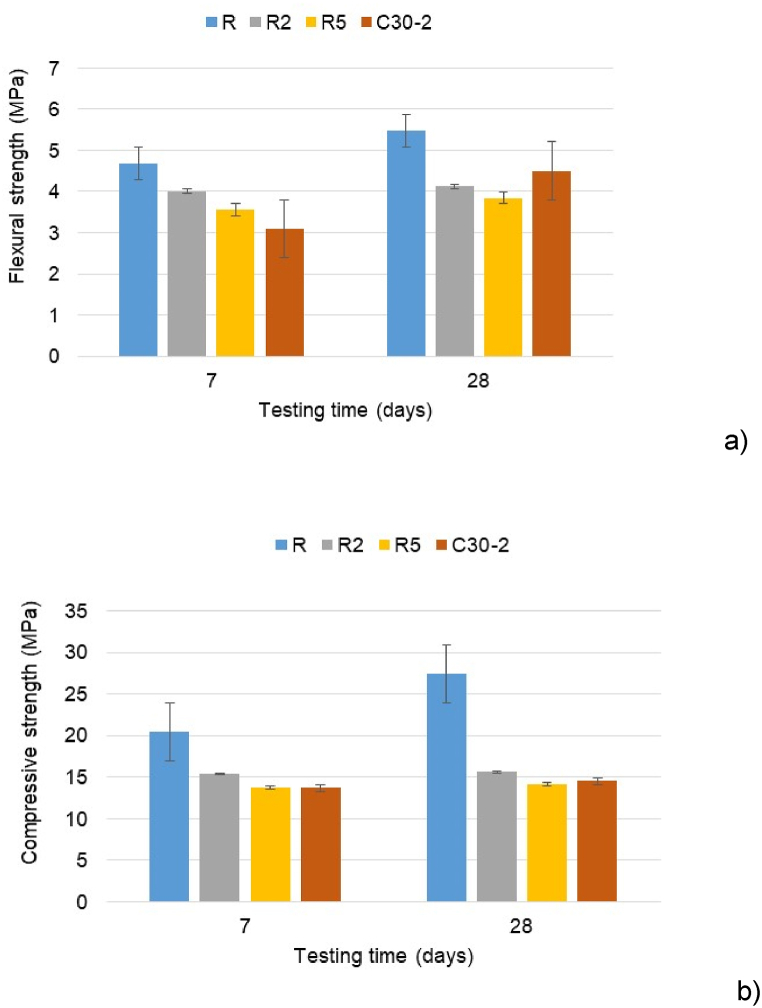
Flexural (a) and compressive strength (b) of reference and mortars with m-PCM.

The presence of PCM negatively influences the mechanical strength of mortars (Fig. 11). The addition of 2 % and 5 % m-PCM into mortars decreased mechanical strength compared to R. The decrease was higher for 5 % m-PCM than that for 2 % m-PCM. The reduction in R5 mortar flexural strength was 23.9 % at 7 days and 29.8 % at 28 days, respectively 14.3 % and 25.6 % for R2 mortar (Fig. 11a). R2 compressive strength diminishing was 24.8 % at 7 days and 42.3 % at 28 days. Similar values were obtained for the R5 mortar (Fig. 11b). A decrease of 7 % in the compressive strength was obtained by Fenollera et al. for every 5 wt% microencapsulated PCM added [66]. The presence of both m-PCM and C-tt slightly influenced the mechanical strength.

Numerous studies identified several causes for the mechanical strength loss of composite materials as a consequence of non-uniform distribution and the damage of the PCM particles [[67], [68], [69], [70], [71], [72]]. It is considered that PCM microcapsules act as voids or defects in concrete due to their extremely low mechanical strength. Furthermore, the porosity of the cement composite increases with the microencapsulated PCM content, affecting the material's density and mechanical properties. Some PCM capsules may be damaged during mixing and leaked paraffin wax which may interfere with or hinder the cement matrix's hydration process (decreasing the formation of calcium silicate hydrate gel). Another reason for the diminishing in mechanical strength is associated with the interfacial transition zone in the mortar that may be disrupted by PCM leaks coating aggregate surfaces.

The mortars were developed for application as interior plasters, meant to be employed as inside wall render finishing. The mortars were categorized according to the results of their compressive strength and compared to standard recommendations, even though they don't require any structural support (Table 6).

**Table 6:** Classification of mortars according to the SR EN 998–1 [73].

Class of Strength	Compressive Strength (MPa)
CS I	0.4–2.5
CS II	1.5–5.0
CS III	3.5–7.5
CS IV	≥6.0

All tested mortars achieved compressive strength over 6 N/mm^2^, which allows the inclusion in the category CS IV.

### 3.7 Relation between the tested properties and composition of mortars

The relationship between tested characteristics of mortars was obtained using experimental results from water absorption, flexural, and compressive strength tests. The flexural strength is estimated using a regression model by using the content of recycled cement. The regression equation presented below had statistical significance.

An efficient technique for assessing the mechanical strength of mortars made using recycled cement was derived from the regression equations, taking into account the Portland cement substitution rate. Equation (7) reveals a small influence of recycled cement content on the flexural strength (f_f_).

(7) f_f_ = 4.41–0.04 ∗ C-tt content for 7 days, R^2^ = 0.91, p < 0.05

Equation (8) shows the relation between flexural strength and compressive strength (f_c_) for 28 days.

(8) f_f_ = 2.46 + 0.11 ∗ f_c_, R^2^ = 0.94, p < 0.05

Equations (9), (10)) present the regression equations with statistical significance for 28-day compressive strength, water absorption (24 h), and the content of recycled cement. The compressive strength is strongly influenced by the content of recycled cement.

(9) f_c_ _28 = -34.51–0.45 ∗ C-tt content +28,30 ∗ W, R^2^ = 0.99, p < 0.05

(10) f_c_ _28 = −66 + 28.16 ∗ f_f_ _7 + 0.34 ∗ C-tt content - 1.87 ∗ f_c_ _7, R^2^ = 0.99, p < 0.05

The relation between flexural strength at 28 days and the other properties is revealed by Equation (11).

(11) f_f_ _28 = 0.39 + 0.95∗ f_f_ _7 + 0.17∗ W – 0.01∗ C-tt content – 0.063∗ f_c_, R^2^ = 0.99, p < 0.05

The findings revealed that replacing Portland cement with recycled cement decreased the mortar's mechanical strength for both testing times. The use of recycled cement affected the properties of mortars, causing a reduction in flexural and compressive strength due to its composition and increased absorption.

### 3.8 SEM

**Fig. 12:**
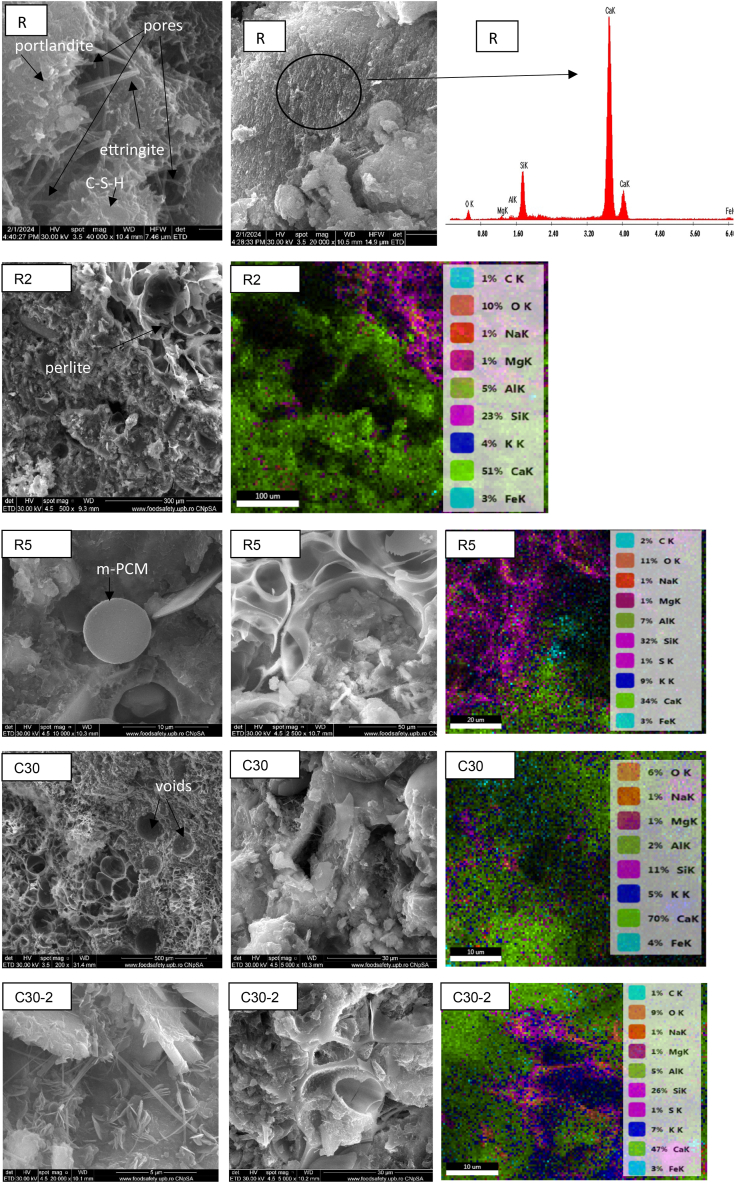
SEM images and elemental analysis for R, R2, R5, C30, and C30-2.

The SEM analyses demonstrate that the cement-based mortar's microstructure is free of cracks, indicating a strong bond between the various components (Fig. 12). Shui et al. [12] noticed that the rehydration products did not have the same morphology as cement hydration products, which led to a cement paste with low properties and high porosity. Shui et al. [74] further demonstrated that the microstructure became denser when the treatment temperature rose. In each mortar sample, the fine rods or needle-like crystals of ettringite were observed. Crumbled foils of C-S-H and hexagonal crystals of Ca(OH)_2_ may be seen as reaction products between calcium silicates and water. The EDAX spectra of the area marked with a circle confirm the presence of significant silicate hydrated with a low amount of Mg, Al, and S. In the case of mortars with C-tt, the hydration processes imply the hydration of Portland cement compounds and rehydration of the previously dehydrated compounds. On the surface of the recycled cement, may be observed the rehydration products with a rough surface and an uneven, fine bundle C-S-H that connects and coats in a honeycomb pattern. A qualitative study of the pores reveals a slight difference between the R and C30 mortar based on SEM morphology. Some noticeable pores can be seen in the mortar that contains 30 % recycled cement. The values of water absorption suggest that pores are less interconnected than those in the R. In the C30 sample, the presence of calcite is notable. The samples with C-tt have a less noticeable microcrystalline character in the SEM images, which explains why the C10, C30, and C50 mortars have lower mechanical strength.

SEM analysis was carried out for the mortars with m-PCM (R2, R5, and C30-2) to assess the distribution of PCM microcapsules within the cementitious mortar matrix and to monitor the microcapsules' condition following their incorporation into the mortar after 28 days.

Microspheres with a diameter under 10 μm correspond to the m-PCM. This value is in the range specified in the product datasheet. Values of approximately 6 μm were measured by Giro-Paloma et al. on slurry PCM called Micronal DS 5001 [75].

The SEM images showed the presence of unbroken microcapsules in the mortars (Fig. 12). Furthermore, the microstructure of the mortars does not seem affected by the presence of spherical m-PCM. The EDAX analysis revealed the element C in mortars with m-PCM. Due to the content in m-PCM, a greater content of C (atomic%) was found for R5. As Fig. 12 showed did not react chemically with cement or aggregate. By comparing the elemental composition (atomic %) provided by EDAX analysis of R2 with R5 was observed a higher content of O, Na, Mg, Mg, Si, and Al. m-PCM The air trapped during the incorporation of the m-PCM particles and the additional water used to maintain the workability of the fresh mortars both contributed to increased porosity [76]. The SEM images revealed an uneven distribution of m-PCM in the hardened mortars. This observation may be explained by the difficulty of incorporating materials with viscous/sticky textures into cement mortars using the same mixing methodology used for powders [77].

## 4 Conclusion and future works

Research concerning the use of m-PCM and recycled cement obtained by calcinating at 550 °C concrete waste (prepared in the laboratory) in expanded perlite mortars is presented in this paper. The experimental results led to the following conclusions.

1. Incorporating treated waste and phase change materials in construction materials is essential for obtaining energy-efficient and sustainable building materials in the context of zero waste challenges.
2. The water absorption rate of mortars, as a function of the square root of time, indicates that capillary absorption increases linearly at early sorptivity stages, with optimal results achieved at 30 % C-tt content. Expanded perlite's pore size distribution significantly influences water absorption, while the addition of m-PCM reduces water absorption due to its nonsorptive nature and increased tortuosity of capillary pathways. The combined use of m-PCM and C-tt generally enhances water resistance compared to standard and lower C-tt content mortars.
3. The experimental results highlight a significant impact of C-tt and m-PCM on the mechanical strength of mortars. Incorporating C-tt and m-PCM decreased flexural and compressive strength, with higher reductions observed at greater C-tt and m-PCM percentages. Despite these reductions, all mortars met the compressive strength requirements for the CS IV category, suitable for non-structural applications.
4. DSC analyses showed that the mortars with m-PCM retain similar transition temperatures as the paraffin, indicating that the paraffin remains a separate solid phase within the mortar. However, the heat of fusion in the mortar samples is lower than expected, suggesting significant paraffin adsorption onto the mortar. Optical microscopy confirms that the shape stability of the mortars is maintained even at temperatures above the paraffin melting point. Also, the SEM analysis confirms the presence of intact microcapsules within the mortar. Additionally, the reference mortar's thermal conductivity is significantly higher than mortars containing recycled cement or m-PCM, with an increasing trend in thermal conductivity observed with rising temperature. This trend underscores the lower thermal conductivity of m-PCM and highlights their potential for enhancing thermal performance in construction materials.

Further research should focus on optimizing the mechanical properties of mortars with microencapsulated phase change materials by improving encapsulation techniques, exploring alternative binders, and conducting long-term durability testing under real-world conditions, such as freeze-thaw cycles and humidity. Energy modeling simulations are part of the author's future development plans for this current study to optimize m-PCM content for different climates, improving building energy efficiency.

## CRediT authorship contribution statement

**Nastasia Saca:** Writing – review & editing, Writing – original draft, Visualization, Validation, Supervision, Resources, Project administration, Methodology, Investigation, Funding acquisition, Conceptualization. **Lidia Radu:** Writing – review & editing, Writing – original draft, Visualization, Investigation. **Răzvan Calotă:** Writing – review & editing, Writing – original draft, Methodology, Investigation, Data curation. **Raul-Augustin Mitran:** Writing – review & editing, Writing – original draft, Validation, Investigation, Conceptualization. **Cosmin Romanițan:** Writing – review & editing, Writing – original draft, Investigation. **Roxana Truşcă:** Writing – review & editing, Investigation. **Carmen Răcănel:** Writing – review & editing, Visualization, Formal analysis. **Ionuț Radu:** Writing – review & editing, Visualization, Investigation.

## Data availability statement

Not applicable.

## Declaration of competing interest

The authors declare that they have no known competing financial interests or personal relationships that could have appeared to influence the work reported in this paper.
